# Contemporaneous Production of Amylase and Protease through CCD Response Surface Methodology by Newly Isolated *Bacillus megaterium* Strain B69

**DOI:** 10.1155/2014/601046

**Published:** 2014-11-12

**Authors:** Rajshree Saxena, Rajni Singh

**Affiliations:** Amity Institute of Microbial Biotechnology, Amity University, Sector 125, Noida, Uttar Pradesh 201303, India

## Abstract

The enormous increase in world population has resulted in generation of million tons of agricultural wastes. Biotechnological process for production of green chemicals, namely, enzymes, provides the best utilization of these otherwise unutilized wastes. The present study elaborates concomitant production of protease and amylase in solid state fermentation (SSF) by a newly isolated *Bacillus megaterium* B69, using agroindustrial wastes. Two-level statistical model employing Plackett-Burman and response surface methodology was designed for optimization of various physicochemical conditions affecting the production of two enzymes concomitantly. The studies revealed that the new strain concomitantly produced 1242 U/g of protease and 1666.6 U/g of amylase by best utilizing mustard oilseed cake as the substrate at 20% substrate concentration and 45% moisture content after 84 h of incubation. An increase of 2.95- and 2.04-fold from basal media was observed in protease and amylase production, respectively. ANOVA of both the design models showed high accuracy of the polynomial model with significant similarities between the predicted and the observed results. The model stood accurate at the bench level validation, suggesting that the design model could be used for multienzyme production at mass scale.

## 1. Introduction

With global population predicted to hit 9 billion people by 2050, the need for additional requirements of agriculture and food will arise throughout the globe [1]. Agricultural wastes constitute a large source of biomass and have potentially detrimental effects both on the environment and human health if not handled and managed properly. Biotechnology offers the best utilization of this waste as alternative substrates in bioprocesses for the production of products as enzymes and food/feed materials using biological entities like microorganisms [2].

Microbial enzymes have wide applications in all industrial to household sector, biotechnological, medicinal, and basic research fields and hold the major share in the global enzyme market [3]. Production of multienzymes from a single fermentation process helps in reducing the cost of the overall production when it comes to industrial application of the enzymes. For efficient and simultaneous production of multienzymes in a single fermentation, bioprocesses with a well-established bioengineering are needed to be developed. Such systems require genetically engineered microorganisms or mixed cultures consisting of different well-designed microbes [4, 5]. However genetic engineering and maintenance of mixed cultures affect the production cost [6]. In this scenario, concomitant production of enzymes, where two or more enzymes are produced in the similar environmental conditions by microorganisms, specifically* Bacillus *sp., can be very well exploited for such multienzyme production without affecting the production cost. This characteristic has been very less explored and very few scientists have mentioned that proteases and amylases are concomitant enzymes. Multienzyme formulations consisting of protease and amylase find applications in production of biofuel, animal feed, personal care products, brewing, detergent, and textile industry [7, 8].

Multienzyme production is a very complex nongrowth associated process with complex patterns of induction and repression resulting from the multisubstrate environment, temperature, pH, moisture content, fermentation time, and inoculum density in solid state fermentation [4, 9, 10]. The interrelation amongst these factors becomes very important aspect to be studied in the multienzyme production. The selection of microorganism also becomes imperative as each microorganism is unique in terms of metabolism and product production pattern, depending mainly on their fermentative, nutritional, physiological, and genetic nature [11]. Thus optimization of production process becomes an important step with particular regard to biotechnology [12]. The time aged classical methods of optimization involve changing one independent variable while maintaining all others at a fixed level. This method is extremely time consuming and does not account for the combined interactions among various physicochemical parameters [13]. Statistical optimization methods, such as Plackett-Burman and Taguchi designs, and response surface methodology have gained interest in the recent years as they overcome the drawbacks of the traditional methods [14, 15]. These methods take into account the interactions of variables in generating process responses and hence are preferred over the conventional optimization methods [16]. These methods allow screening of significant factors affecting a process from a large number of process variables and studying their interactive effect on a single or multiresponse [17]. RSM (response surface methodology) designs evaluate relationships between one or more responses and their interactive effect on a process resulting in the optimum required conditions [18, 19].

The present study exploits the unique property of concomitant production of protease and thermostable amylase by a newly isolated and identified* Bacillus megaterium* B69 strain. A statistical model was developed employing Plackett-Burman and a quadratic central composite design in response surface methodology for obtaining the optimized conditions for multienzyme production in solid state fermentation utilizing agro-industrial residues.

## 2. Materials and Methods

### 2.1. Microorganism

A newly isolated* Bacillus* sp. producing protease and amylase concomitantly was selected from microbial culture collection available in the laboratory.

### 2.2. Molecular Identification of the Strain

#### 2.2.1. DNA Extraction

The genomic DNA of the selected strain was extracted by Moore et al.'s [20] modified phenol chloroform extraction method.

#### 2.2.2. PCR Amplification and Sequencing of 16S* r*DNA

The amplification reaction was performed in a 50 *μ*L volume by mixing template DNA (2 *μ*L), 1 *μ*L (75 pmol/*μ*L) forward primer (5′ AGAGTTTGATCCTGGCTCAG 3′), 1 *μ*L (75 pmol/*μ*L) reverse primer (5′ TACGGCTACCTTGTTACGACTT 3′), 25 *μ*L mastermix (1X, G-Biosciences) containing* Taq* polymerase, and PCR reaction buffer and dNTPs. DNA amplification was done in a DNA thermal cycler (Mastercycler pro, Eppendorff) with the following temperature profile: initial denaturation at 94°C for 5 min, 40 cycles of denaturation at 94°C for 30 sec, annealing temperature at 50°C for 30 sec, and extension at 72°C for 1 min, with a final extension at 72°C for 10 min. The amplified product along with DNA molecular weight markers was run on a 0.8% agarose gel mixed with ethidium bromide at a constant voltage (60 v) and visualized in gel documentation system (InGenius3, Synegene). Amplified DNA product was eluted from agarose gel using Qiagen gel elution kit as per the manufacturer's instructions and protocol. The pure eluted amplified DNA product was sequenced using Automated ABI 3100 Genetic Analyzer.

#### 2.2.3. Phylogenetic Analysis and Strain Identification

The obtained 16S* r*DNA sequence was subjected to nucleotide blast (blastn) at NCBI to retrieve homologous sequences and identify the strain to the generic level. The multiple sequences were aligned using CLUSTALW2, the multiple sequence alignment program from EMBL-EBI, UK, and the phylogenetic tree was constructed through neighbor-joining method in Phylip and viewed using TreeView program [21].

### 2.3. Concomitant Production of Amylase and Protease in Solid State Fermentation

#### 2.3.1. Substrate

Six types of agro-industrial waste, that is, gram husk, wheat bran, rice bran, corn husk, mustard oilseed cake, and soybean cake, were procured from the local mills and processed to obtain a uniform size of about 2–4 mm.

#### 2.3.2. Solid State Fermentation

The selected strain was inoculated in nutrient broth (containing (g/l) peptone-5; NaCl-5; beef extract-3) and incubated at 37°C for 24 h at 120 rpm to obtain a standard inoculum (0.6 O.D).

The SSF experiments were conducted in 250 mL Erlenmeyer flasks containing solid substrate material supplemented with distill water containing soluble mineral salts K_2_HPO_4_, KH_2_PO_4,_ NaCl, MgSO_4_ *·*7H_2_O, NaNO_3_, and CaCl_2_ in varying concentrations. The contents of the flasks were mixed thoroughly, autoclaved at 121°C for 15 min at 15 lbs, cooled, inoculated with the prepared inoculum, and incubated at 37°C for the desired period. The fermentation media was centrifuged at 10000 rpm for 10 min. The supernatant was taken as the crude enzyme and assayed for the activity.

### 2.4. Enzyme Assay

Protease activity was measured using casein as substrate [22]. One unit of protease activity was defined as the amount of enzymes required to liberate 1 *μ*g tyrosine per mL in 1 min under the experimental conditions used.

Estimation of amylase activity was carried out according to Miller's DNSA method [23]. One unit of enzyme activity is defined as the amount of enzymes, which releases 1 *μ*g of reducing sugar as glucose per minute, under the assay conditions. The experiments were carried out in triplicates and standard error was calculated.

### 2.5. Optimization Studies

#### 2.5.1. Selection of Substrate

Among the six types of agro-residues taken, mustard oilseed cake was best utilized for concomitant protease and amylase production by the selected bacterial strain. Hence it was selected for further optimization studies.

#### 2.5.2. Statistical Optimization of Production Parameters

Two-step statistical techniques were employed for optimization of enzyme production parameters. In the first step significant variables that affected the production were identified by Plackett-Burman design, while in the second step, optimization of the screened variables was performed by central composite design. Design Expert 8.0.2.0 (Stat-Ease, Inc., Minneapolis, MN, USA) was used to design and analyze the experiments.

#### 2.5.3. Plackett-Burman Design for Primary Screening of Factors

The Plackett-Burman design [24] is a 2-factorial design that mathematically computes, evaluates, and screens out the most significant media components that influence enzyme production from a large number of factors in one experiment, allowing insignificant factors to be eliminated to obtain a minimized number of variables. This is based on the first order model given by

(1) $$\begin{array}{c} E\left(x_{i}\right)=\frac{2\left[\sum_{}^{}\left(M_{i}+\right)-\left(M_{i}-\right)\right]}{N}, \end{array}$$

where *E*(*x*_*i*_) is the concentration effect of the tested variable, *M*_*i*_+ and *M*_*i*_− are the total production from the trials where the measured variable (*x*_*i*_) was examined in two levels, (−) for low level and (+) for high level, and *N* is the number of trials. The 12-run PB design was used to study ten physicochemical factors, namely, substrate concentration, inoculum size, moisture content, incubation time, and trace elements K_2_HPO_4_, KH_2_PO_4,_ NaCl, MgSO_4_ *·*7H_2_O, NaNO_3_, and CaCl_2_.

#### 2.5.4. Centre Composite Design (CCD) for RSM

Threefactors, namely, substrate concentration, moisture content, and incubation time, were found to significantly affect the enzyme production as Plackett-Burman design analysis. Central composite experimental design in RSM was used to obtain an optimum combination of the three selected variables, where each factor is varied over 5 levels (alpha = 1.682), 2 axial points (+ and − alpha), 2 factorial points (+ and −1), and 1 centre point resulting in a total of 20 experiments. The design summary for two responses, protease activity and amylase activity, is represented in Table 4.

#### 2.5.5. Statistical Analysis and Modelling

The results obtained in the experimental runs were subjected to analysis of variance (ANOVA) in CCD. A second-order polynomial equation (2) can be used to represent the function of the interacting factors to calculate the predicted response.

(2) Y=β0+β1X1+β2X2+β3X3+β11X12+β22X22+β33X32 +β12X1X2+β13X1X3+β23X2X3,

where *Y* is the measured response, *β*_0_ is the intercept term, and *β*_1_, *β*_2_, and *β*_3_ are linear coefficients, *β*_11_, *β*_22_, and *β*_33_ are quadratic coefficients, *β*_12_, *β*_13_, and *β*_23_ are interaction coefficients, and *X*_1_, *X*_2_  and *X*_3_ are coded independent variables.

### 2.6. Validation of the Experimental Model at Bench Level

The factors obtained after Plackett-Burman and CCD were checked for their accuracy for the two responses. The statistical model was validated with respect to all the three variables within the design space. A random set of 6 experimental combinations was used to study protease and amylase production under the experimental conditions.

## 3. Results

### 3.1. Identification of the Selected Strain

#### 3.1.1. Biochemical Characterization

The morphological, microscopic, and biochemical characteristics of the bacterial strain are represented in Table 1. The strain was observed as round medium-sized white colonies with defined margin and slimy texture that grew aerobically. Microscopic study revealed spore forming and gram positive rods.* Bacillus* represents the large genus in family Bacillaceae that are gram-positive rods and form a unique, dormant, tough, and nonreproductive resting cell called endospore [25]. The motility test showed a motile organism. Most of the* Bacillus* sp. (except* B. anthracis* and* B. cereus* subsp.* mycoides*) are known to be motile [26].

The selected strain was able to utilize citrate, starch, exhibited catalase and gelatinase activities, and converted nitrate to nitrite. It utilized various sugars with gas production. However, it was found to be indole, MR, and VP negative and did not show oxidase activity. On the basis of Bergey's Manual of Determinative Bacteriology, the phenotypical characteristics suggested that the selected strain belongs to genus* Bacillus*.

#### 3.1.2. 16S* r*DNA Gene Sequencing and Strain Identification

The blast studies performed with sequence of the amplified 16s* r*DNA showed that the strain exhibited 93.0–99.0% similarity with different* Bacillus* species and 99% similarity with various strains of* B. megaterium* and* B. aryabhattai*. Thus on the basis of biochemical and molecular studies the* Bacillus* strain was identified as a new* Bacillus megaterium* strain B69.

#### 3.1.3. Phylogenetic Analysis

**Figure 1:**
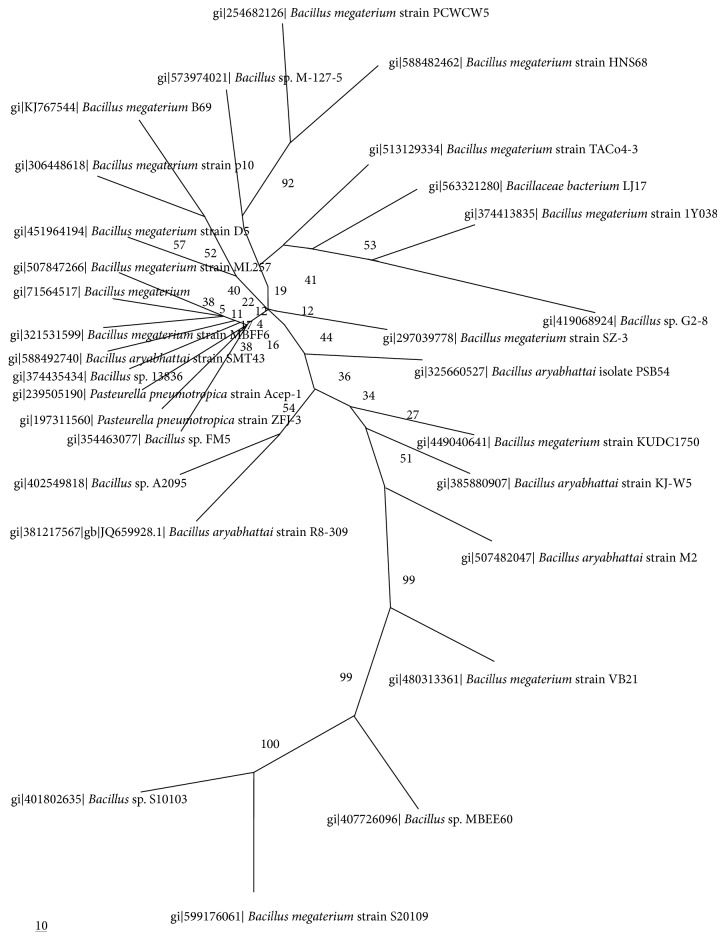
Phylogenetic tree showing evolutionary relationships between strain* Bacillus cereus* B80 and other closely related* Bacillus* species.

The phylogenetic tree showed the detailed evolutionary relationships between the newly identified strain* Bacillus megaterium* B69 and other closely related* Bacillus* species mainly* B. megaterium* and* B*.* arayabhattai* and demonstrated a distinct phylogenetic position of this strain within the genus (Figure 1).

#### 3.1.4. Nucleotide Sequence Accession Number

The GenBank/NCBI accession number of the strain* Bacillus megaterium* B69 is* KJ767544*.

### 3.2. Optimization Studies

#### 3.2.1. Selection of the Solid Substrate

**Figure 2:**
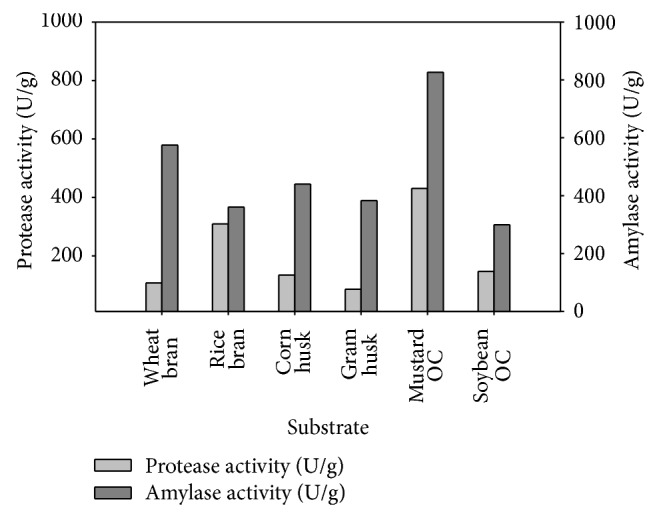
Protease and amylase production with different agro-residues.

Maximum concomitant production of protease and amylase by the selected* Bacillus megaterium* B69 strain was observed with mustard oilseed cake. Rice bran also produced significant amount of protease, but wheat bran, corn husk, gram husk, and soybean oil cake exhibited less protease production (Figure 2). However amylase production was significantly good with all agro residues. Owing to the cost, availability, and maximum units of enzyme obtained, mustard oilseed cake was selected as substrate for further optimization.

#### 3.2.2. Plackett-Burman Design

**Figure 3:**
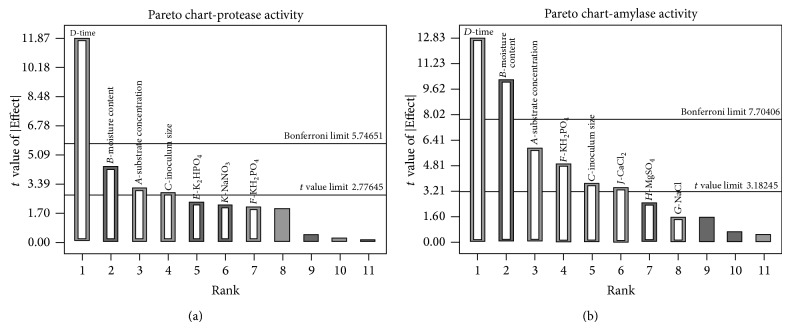
Pareto chart showing the relative effect of various factors on protease and amylase Production.

Plackett-Burman design was employed for screening the significant variables amongst the ten parameters taken for the enzyme production in solid state fermentation. The design matrix and the corresponding responses are shown in Table 2.  Table 3(a) represents the *E*(*x*_*i*_) value of the variables investigated. A large *E*(*x*_*i*_) coefficient, either positive or negative, indicates a large impact on response, while a coefficient close to zero indicates little or no effect (Figure 3). The results show that substrate concentration, moisture content, and time exhibited maximum *E*(*x*_*i*_) value (+ or −) for both protease and amylase production; hence, these were selected for second level optimization in CCD. Inoculum size, KH_2_PO_4_, and NaCl exhibited positive effect; hence, they were taken at their maximum limit. MgSO_4_, CaCl_2_, and K_2_HPO_4_ exhibited negative *E*(*x*_*i*_) values; hence, they were taken in their lower limits. NaNO_3_ exhibited high negative value; hence, it was eliminated.

The adequacy of the Plackett-Burman design was calculated via ANOVA (Table 3(b)). The Model *F* value of 27.52 for protease production and 45.31 for amylase production implies the model is significant, with only 0.32 and 0.48% chances in protease and amylase production, respectively, that this large “Model *F*-Value” could occur due to noise. Values of “Prob > *F*” less than 0.0500 indicate model terms are significant. In the designed model *A*, *B*, *C*, and *D*, for protease production and *A*, *B*, *C*, *D*, *F*, and *J*, for amylase production, were found to be significant model terms. Degrees of freedom for evaluation of the model shows a lack of fit 1 that ensures a valid lack of fit test. The Pred *R*-Squared for both protease and amylase production is in reasonable agreement with the Adj *R*-Squared (Table 3(c)). Adeq Precision (measure of signal to noise ratio) is 15.365 and 17.662 (a ratio greater than 4 is desirable) for protease and amylase production, respectively, which indicates an adequate signal. This model can be used to navigate the design space.

#### 3.2.3. Central Composite Design

Three significant factors, substrate concentration, moisture ratio, and time, were selected for second step of optimization through CCD in response surface methodology on the basis of the results of Plackett-Burman design. A statistical model consisting of 20 runs with three significant variables was designed. The design model with corresponding responses of actual and predicted values is represented in Table 4.

#### 3.2.4. Statistical Analysis of Variance (ANOVA) of CCD

The statistical testing of the model for the two-response protease and amylase production was done by Fisher's statistical test for analysis of variance (ANOVA) and the results are shown in Table 5. The Model *F* value of 162.08 and 33.62 for protease and amylase production, respectively, implies the model is significant with only 0.01% chance that a Model *F* value this large could occur due to noise. Values of “Prob > *F*” less than 0.0500 indicate model terms are significant. In the designed model, for protease production *A*, *B*, *C*, *AB*, *BC*, *A* ^2^, *B* ^2^, and *C* ^2^ are significant model terms, while for amylase production *A*, *B*, *C*, *A* ^2^, *B* ^2^, and *C* ^2^ are significant model terms. The “Lack of Fit *F* value” of 4.21 and 2.94 for observed for protease and amylase production, respectively, implies the that the Lack of Fit is not significant relative to the pure error. There is 7.02% and 13.10% chance for protease and amylase production, respectively, that a “Lack of Fit *F* value” this large could occur due to noise. The nonsignificant lack of fit is good as it fits the model.

The regression equation coefficients were calculated and the data were fitted into a second-order polynomial equation for the two responses, represented in terms of coded factors as follows:

(3) Protease  Activity=+1249.19+133.71∗A+80.58∗B+112.14∗C −57.04∗AB−0.024∗AC−62.62∗BC−209.73∗A2 −79.56∗B2−297.74∗C2.Amylase  Activity=+1606.93+128.14∗A+103.71∗B+273.04∗C −13.72∗AB+30.92∗AC−33.39∗BC−402.22∗A2 −147.20∗B2−383.13∗C2,

where *A* is substrate concentration, *B* is moisture content, and *C* is time.

The regression equation obtained from the ANOVA (Table 5) showed that the multiple correlation coefficients (*R* ^2^) 0.9931 and 0.9680 for protease and amylase activity, respectively, indicate fitness of the model. Also, the Pred *R*-Squared values are in reasonable agreement with the Adj *R*-Squared for both the responses. Adeq Precision of 36.329 and 16.128 for protease and amylase production indicates an adequate signal. This model can be used to navigate the design space.

**Figure 4:**
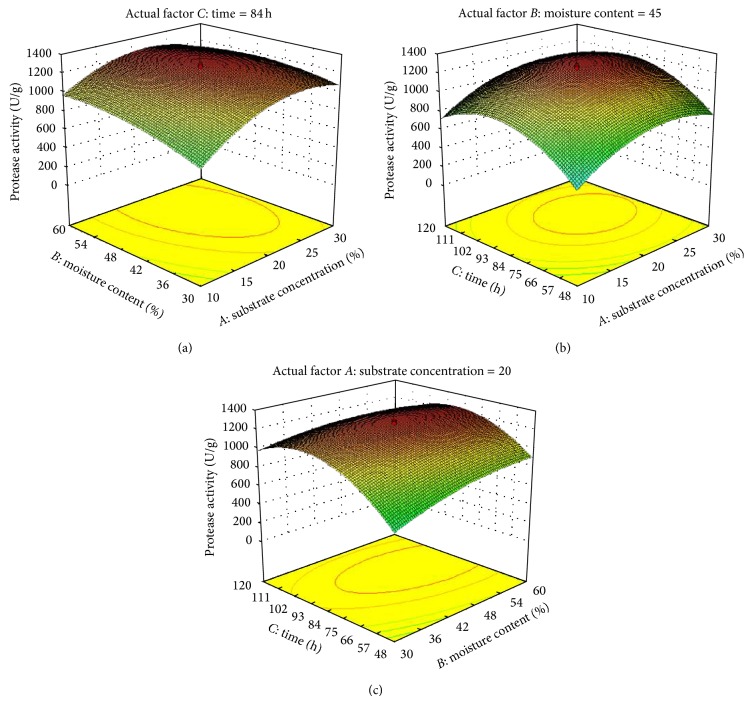
Contour plots for protease production as a function of the interactions of two variables by keeping the other at centre level: (a) interactions of substrate concentration and time with moisture content at 45%, (b) interactions of substrate concentration and with time at 84 h, and (c) interactions of moisture content and time with substrate concentration at 20%.

**Figure 5:**
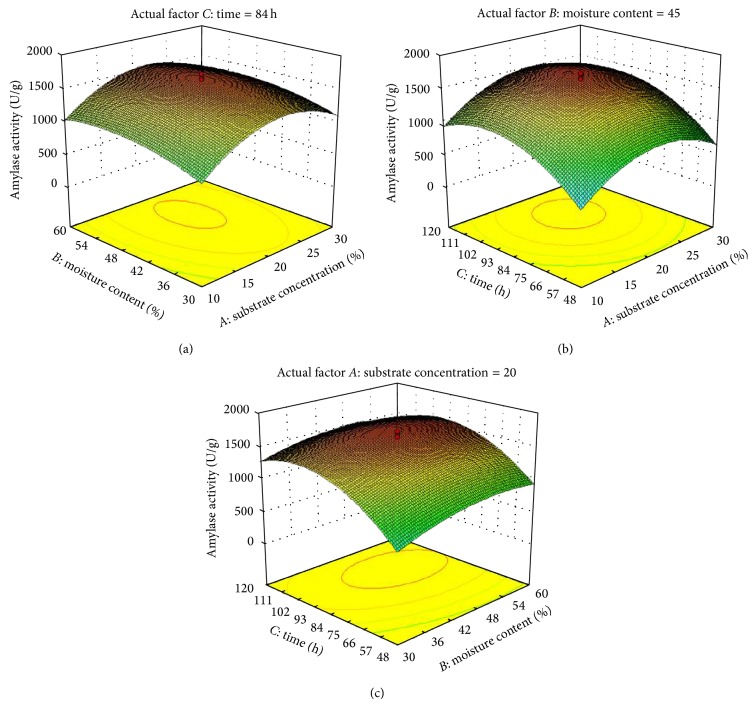
Contour plots for amylase production as a function of the interactions of two variables by keeping the other at centre level: (a) interactions of substrate concentration and time with moisture content at 45%, (b) interactions of substrate concentration and time at 84 h, and (c) interactions of moisture content and time with substrate concentration at 20%.

Three-dimensional response surface contour graphs were plotted with the responses (protease and amylase production) on the *Z*-axis against any two independent variables, while maintaining one variable at its optimal level. The interaction between coded variables and responses is more accurately understood by these of surface plots.  Figure 4(a) shows an increase in protease production was observed substrate concentration and time increase but further increase in these two factors resulted in decrease of the response, when moisture content was maintained at its optimum. Similarly the enzyme production increased by increasing the substrate concentration and moisture content (Figure 4(b)) and moisture content and time (Figure 4(c)), while keeping time and substrate concentration constant, respectively. But in both the cases the response decreased after an optimal level of conditions was reached. Similar results were observed with the three factors for amylase production (Figure 5). All the plots (Figures 4 and 5) exhibit a fairly strong degree of curvature of 3D surface where the optimum level of the variable for the response can easily be determined.

Thus the maximum protease and amylase production were 1280.2 and 1725.8 U/g after 84 h when the substrate concentration was 20% and moisture ratio was 45%.

### 3.3. Validation of the Statistical Design Model

The results for the validation experiment show that the experimental values for the two responses stand in close agreement with the predicted values. The maximum protease and amylase activity were observed at 20% substrate concentration and 45% moisture content after 84 h of incubation (Table 6). The results verify the accuracy of the model.

## 4. Discussion

The most significant outcome of the present study is multi enzyme production from a single fermentation system, lowering the cost of production. The use of cheap and readily available agricultural residue as mustard oilseed cake as the substrate in solid state fermentation also lowers the cost of the production. Generally, after production from cheap sources, purification of the enzymes becomes a time consuming and expensive step, thereby affecting the overall cost of the process. Stability of two enzymes with each other also becomes an issue if they are synthetically mixed for a process. However, in the concomitant production less manipulation is required for the maintenance and stability of the enzymes. In our study as amylases is produced along protease, it is protease resistant by virtue of its production. This stability of amylases could have wide application in various biotechnological fields. The concomitant production of protease and amylase aims at the industries as food and feed, pharmaceuticals, detergent, and so forth, where these enzymes can be used in a synergistic system.

The use of Plackett-Burman and centre composite design in RSM for optimization of production factors resulted in enhancement of 2.95- and 2.04-fold in protease and amylase production, respectively. The model equation (3) indicates that substrate concentration (*A*) and time (*C*) had a significant effect (*P* < 0.0001) on responses 1 and 2 with largest coefficients. The statistical analysis (ANOVA) of both the designs exhibits a high precision of the polynomial model and a high degree of fitting between the predicted and the experimental data for both the responses. This great similarity between the predicted and the observed results validates the accuracy and applicability of the model in the optimization processes.

## 5. Conclusion

The unique property of concomitant production of protease and amylase by the* Bacillus* sp. used in the present study provides a potential for biotechnological applications of the strain. Multienzyme complexes include two or more enzymes working in close association and synergistically provide a multitude of products by degradation of complex substrates with higher efficiency than individual enzymes [27]. The validation and accuracy of the statistical models establish that the present study could be exploited for various industrial and biotechnological applications.

## Figures and Tables

**Table 1:** Morphological and biochemical tests performed for identification of selected bacterial isolate.

Morphological tests	
Grams staining	+
Cell shape	Rods
Spore formation	+
Motility	+
Biochemical tests	
Indole production	−
Methyl red	−
Voges-Proskauer	−
Citrate utilization	+
Oxidase test	−
Catalase test	+
Starch hydrolysis	+
Nitrate reduction	+
Casein hydrolysis	+
Cellulase hydrolysis	+
Gelatin hydrolysis	+
Glucose utilization	+
Lactose utilization	+

**Table 2:** Plackett-Burman design and responses.

Std run	Factor 1	Factor 2	Factor 3	Factor 4	Factor 5	Factor 6	Factor 7	Factor 8	Factor 9	Factor 10	Response 1	Response 2
	*A*: substrate	*B*: moist content	*C*: inoculum size	*D*: time	*E*: K_2_HPO_4_	*F*: KH_2_PO_4_	*G*: NaCl	*H*: MgSO_4_	*J*: CaCl_2_	*K*: NaNO_3_	Protease activity	Amylase activity
	%	%	%	h	%	%	%	%	%	%	U/g	U/g
1	10	75	3	72	1	1	0.1	0.1	0.01	1	311.87	868.76
2	5	75	5	24	1	1	0.5	0.1	0.01	0.01	74.27	354.59
3	10	50	5	72	0.1	1	0.5	1	0.01	0.01	697.35	1775.04
4	5	75	3	72	1	0.1	0.5	1	0.05	0.01	273.19	542.51
5	5	50	5	24	1	1	0.1	1	0.05	1	79.57	638.55
6	5	50	3	72	0.1	1	0.5	0.1	0.05	1	475.02	1485.09
7	10	50	3	24	1	0.1	0.5	1	0.01	1	75.05	471.46
8	10	75	3	24	0.1	1	0.1	1	0.05	0.01	85.67	429.88
9	10	75	5	24	0.1	0.1	0.5	0.1	0.05	1	120.86	486.66
10	5	75	5	72	0.1	0.1	0.1	1	0.01	1	296.17	451.95
11	10	50	5	72	1	0.1	0.1	0.1	0.05	0.01	530.86	1420.33
12	5	50	3	24	0.1	0.1	0.1	0.1	0.01	0.01	73.71	391.6

**(a):** 

Variable	Protease activity				Amylase activity		
	Component	*M*_*i*_+	*M*_*i*_−	*E*[*x*_*i*_]	*M*_*i*_+	*M*_*i*_−	*E*[*x*_*i*_]
*A *	Substrate concentration	1821.66	1271.93	91.62	3752.13	2564.29	197.97
*B *	Moisture content	1162.03	1931.56	−128.26	2134.36	4182.07	−341.29
*C *	Inoculum size	1799.08	1294.51	84.09	3527.12	2789.3	122.97
*D *	Time	2584.46	509.13	345.89	4443.68	1872.75	428.49
*E *	K_2_HPO_4_	1344.81	1748.78	−67.33	3096.21	3220.22	−20.67
*F *	KH_2_PO_4_	1723.75	1369.84	58.99	3651.92	2664.51	164.58
*G *	NaCl	1715.74	1377.85	56.32	3315.36	3001.07	52.38
*H *	MgSO_4_	1507	1586.59	−13.27	2909.39	3407.04	−82.94
*I *	CaCl_2_	1565.17	1528.42	6.13	3503.02	2813.4	114.94
*J *	NaNO_3_	1358.54	1735.05	−62.75	3002.47	3313.96	−51.91

**(b):** 

Response	Source	Sum of squares	df	Mean square	*F* value	*P* value Prob > *F*	
Protease activity	Model	4.905*E* + 005	7	70073.44	27.52	0.0032	Significant
Amylase activity	Model	1.213*E* + 006	8	1.516*E* + 005	45.31	0.0048	Significant

**(c):** 

	Std. Dev.	Adeq precision	*R*-Squared	Adj *R*-Squared	Pred *R*-Squared
Protease activity	50.46	15.365	0.9797	0.9441	0.8169
Amylase activity	57.85	17.662	0.9918	0.9699	0.8687

**Table 4:** Central composite design matrix for the experimental design and predicted responses for protease activity.

Std	Factor 1	Factor 2	Factor 3	Response 1		Response 2	
	*A*: substrate concentration	*B*: moisture content	*C*: time	Protease activity (U/g)		Amylase activity (U/g)	
	%	%	h	Actual	Predicted	Actual	Predicted
1	10	30	48	246.15	216.05	204.03	153.31
2	30	30	48	625	597.6	296.33	375.19
3	10	60	48	630.19	616.55	480.9	454.95
4	30	60	48	737.69	769.92	560.9	621.95
5	10	30	120	595.58	565.61	661.67	704.32
6	30	30	120	931.15	947.06	920.24	1049.88
7	10	60	120	685.96	715.63	847.57	872.4
8	30	60	120	836.54	868.91	1008.67	1163.09
9	3.18	45	84	403.85	431.13	198.33	253.79
10	36.82	45	84	911.35	880.86	886.9	684.8
11	20	19.77	84	845	888.64	1085.37	1016.18
12	20	70.23	84	1206.54	1159.69	1442.49	1365.02
13	20	45	23.45	194.23	218.46	51.69	64.08
14	20	45	144.55	623.08	595.64	1141.5	982.46
15	20	45	84	1218.46	1219.19	1525.84	1606.93
16	20	45	84	1240.2	1239.21	1462.77	1606.93
17	20	45	84	1242.12	1239.21	1666.31	1606.93
18	20	45	84	1220.05	1239.21	1625.82	1606.93
19	20	45	84	1242.94	1239.21	1643.57	1606.93
20	20	45	84	1251	1239.21	1592.13	1606.93

**(a):** 

Response	1 Protease activity				2 amylase activity				Protease & amylase activity	
Source	Sum of squares	Mean square	*F* value	*P* value Prob > *F*	Sum of squares	Mean square	*F* value	*P*-value Prob > *F*	df	
Model	2.281*E* + 006	2.534*E* + 005	162.08	<0.0001	5.538*E* + 006	6.154*E* + 005	33.62	<0.0001	9	Significant
*A*-subs Conc.	2.441*E* + 005	2.441*E* + 005	150.94	<0.0001	2.242*E* + 005	2.242*E* + 005	12.25	0.0057	1	
*B*-moisture	88686.05	88686.05	54.83	<0.0001	1.469*E* + 005	1.469*E* + 005	8.03	0.0178	1	
*C*-time	1.717*E* + 005	1.717*E* + 005	106.17	<0.0001	1.018*E* + 006	1.018*E* + 006	55.63	<0.0001	1	
*AB*	26031.48	26031.48	16.09	0.0025	1506.14	1506.14	0.082	0.7801	1	
*AC*	4.623*E* − 003	4.623*E* − 003	2.858*E* − 006	0.9987	7648.89	7648.89	0.42	0.5325	1	
*BC*	31370.31	31370.31	19.39	0.0013	8918.14	8918.14	0.49	0.5011	1	
*A* ^2^	6.339*E* + 005	6.339*E* + 005	391.89	<0.0001	2.331*E* + 006	2.331*E* + 006	127.39	<0.0001	1	
*B* ^2^	91212.08	91212.08	56.39	<0.0001	3.122*E* + 005	3.122*E* + 005	17.06	0.0020	1	
*C* ^2^	1.278*E* + 006	1.278*E* + 006	789.83	<0.0001	2.115*E* + 006	2.115*E* + 006	115.59	<0.0001	1	
Residual	16174.90	1617.49			1.830*E* + 005	18301.72			10	
Lack of fit	13073.08	2614.62	4.21	0.0702	1.365*E* + 005	27308.68	2.94	0.1310	5	Not significant
Pure error	3101.82	620.36			46473.76	9294.75			5	
Cor total	2.345*E* + 006				5.721*E* + 006				19	

**(b):** 

	Std. dev.	Adeq precision	*R*-squared	Adj *R*-squared	Pred *R*-squared
Protease	39.54	36.329	0.9931	0.9869	0.9551
Amylase	135.28	16.128	0.9680	0.9392	0.8072

**Table 6:** Validation of the design model.

Run	Factor 1	Factor 2	Factor 3	Response 1 protease activity (U/g)		Response 2 amylase activity (U/g)	
	*A*: substrate concentration (%)	*B*: moisture ratio	*C*: time (h)	Experimental	Predicted	Experimental	Predicted
1	10	60	48	618.8	630.57	426.67	451.75
2	30	60	120	742.5	734.02	1104.7	1116.39
3	20	19.77	84	817.9	836.01	1034	1067.11
4	20	45	144.55	626.4	638.06	1012.7	1010.72
5	20	45	84	1286.6	1220.2	1615.4	1627.87
